# Mysteries of icosahedral quasicrystals: how are the atoms arranged?

**DOI:** 10.1107/S2052252516009842

**Published:** 2016-06-27

**Authors:** Tsutomu Ishimasa

**Affiliations:** a Division of Applied Physics, Graduate School of Engineering, Hokkaido University, Sapporo 060-8628, Japan

**Keywords:** quasicrystals, superspace crystallography, structure analysis, phasons, X-ray diffuse scattering

## Abstract

Higher-dimensional structure analysis of quasicrystals is now possible. Yamada *et al.* [*IUCrJ* (2016), **3**, 247–258] have solved the atomic structure of icosahedral ScZn_7.33_ including the characteristic imperfections.

A quasicrystal is a solid or soft matter that exhibits diffraction symmetries that are not allowed for a periodic crystal. Quasicrystals are classified into several types (pentagonal, octagonal, decagonal, dodecagonal and icosahedral) according to their symmetry. The arrangement of atoms in a quasicrystal does not obey periodicity, but obeys a special rule, quasiperiodicity, over a long range. The quasiperiodicity can be related to a geometric progression, the common ratio of which is an irrational number, for example, the golden ratio. This property can be seen in Fig. 1 ▸, which shows the electron diffraction pattern of an icosahedral Sc–Zn–Mg quasicrystal observed along the fivefold axis. Since the discovery of quasicrystals by Shechtman *et al.* (1984 ▸), the problem of how atoms are arranged in an actual quasicrystal has attracted a great deal of interest from crystallographers. Like the case of a periodic crystal, it is now considered that the structure of a quasicrystal is understood firstly from the ideal or average structure, and secondly from deviations from it. In the case of an icosahedral quasicrystal, Amman tiling (a three-dimensional version of the famous Penrose tiling) is available as a starting model for the ideal structure. As deviations, a quasicrystal may have defects related to tile or cluster rearrangement, which is a phason degree of freedom that is not present in a usual crystal (Socolar *et al.*, 1986 ▸). Besides the structure, scientists have also tackled the following problems: Why does such a strange structure form as a stable phase? How does the quasicrystal grow during a relatively short period? What are the physical properties inherent to a quasicrystal?

In a recent **IUCrJ** paper by Yamada *et al.* (2016 ▸), entitled ‘Atomic structure and phason modes of the Sc–Zn icosahedral quasicrystal’, the atomic structure of a Tsai-type quasicrystal has been studied by both synchrotron radiation diffraction and diffuse scattering experiments with the help of state-of-the-art higher-dimensional analysis (Duneau & Katz, 1985 ▸; Elser, 1986 ▸; Yamamoto, 1996 ▸). The i-ScZn_7.33_ quasicrystal (Goldman *et al.*, 2013 ▸) belongs to the same structure type as i-YbCd_5.7_ (Tsai *et al.*, 2000 ▸), the structural model of which was proposed by Takakura *et al.* (2007 ▸). It was shown that the i-ScZn_7.33_ quasicrystal consists of two types of building blocks: one is a rhombic triacontahedron, and the other a rhombus called a ‘double Friauf polyhedron’. The triacontahedron consists of five shells arranged in a concentric manner: the innermost tetrahedron, the second dodecahedron, the third icosahedron, the fourth icosidodecahedron and the outermost triacontahedron. This is called a Tsai-type cluster. The triacontahedra are located at so-called 12-fold vertices in the Amman tiling (Henley, 1986 ▸), and the remaining space is filled by the double Friauf polyhedra. They are arranged quasiperiodically and form an ideal structure. The work by Yamada *et al.* (2016 ▸) also provides new information on the deviations. The first is chemical disorder between Sc and Zn at the rare-earth sites; this explains the difference in alloy compositions between i-YbCd_5.7_ and i-ScZn_7.33_. The second is the existence of a Zn_4_ tetrahedron centered at the Tsai-type cluster, and the third is long-wavelength phason fluctuations that cause characteristic diffuse scattering.

The Tsai-type cluster corresponds to the initial term in the geometric progression. The most unusual feature of the Tsai-type cluster is a mismatch between the Zn_4_ tetrahedron and the outer icosahedral shells. The work by Yamada *et al.* (2016 ▸) proposes a clever way of solving this problem. The distorted Tsai-type clusters, each of which includes an oriented Zn_4_ tetrahedron, are arranged systematically to satisfy the icosahedral symmetry as a whole. Then there is no symmetry breaking in the diffraction patterns. It is impressive that this structure model was derived from the intensity data using higher-dimensional analysis. On the other hand, in the case of a cubic approximant crystal formed in the same Sc–Zn alloy, it is known that the central Zn_4_ tetrahedron behaves as a single molecule and exhibits picosecond motion, or dynamical flexibility, above approximately 150 K (Euchner *et al.*, 2012 ▸). In both cases, the cluster does not satisfy the icosahedral symmetry from either local or time viewpoints. However, nature knows at least two methods to avoid this difficulty in the initial term. One is spatial averaging; the other is time averaging. It is necessary to clarify which model is best for individual quasicrystals.

In their work, Yamada *et al.* (2016 ▸) observed a significant amount of diffuse scattering elongated along the threefold direction, and also an intensity reduction at weaker reflections. These observations indicate the occurrence of phason fluctuations. Using hydro­dynamic theory, an analysis of the diffuse scattering intensity indicated that the i-ScZn_7.33_ quasicrystal is situated at the limit of the threefold instability. All deviations, namely the chemical disorder, distortion of clusters and long-wavelength phason fluctuations observed here, may be more or less common in many icosahedral quasicrystals. Although their mutual and atomic scale relationship is not understood yet, it is sure that the deviations contribute to stabilize the quasicrystal by increasing the entropy. This consideration seems to support the scenario of an entropy-stabilized model rather than a quasperiodic model as the ground state. However, the problem of the stabilization mechanism is not so simple, because the degree of structural perfection of a quasicrystal strongly depends on the alloy system as demonstrated in this work: i-Sc–Zn–Mg exhibits many more weaker reflections than i-ScZn_7.33_, and also weaker diffuse scattering intensity. Accordingly, by finding a proper combination of elements, there is still a hope of finding the perfect quasicrystal forming as a ground state.

In a recent study, it has been reported that the i-Yb–Au–Al quasicrystal, isostructural to i-YbCd_5.7_, shows unique quantum criticality (Deguchi *et al.*, 2012 ▸). In order to study such physical properties, a detailed structural analysis like the one performed in Yamada *et al.* is certainly necessary.

## Figures and Tables

**Figure 1:**
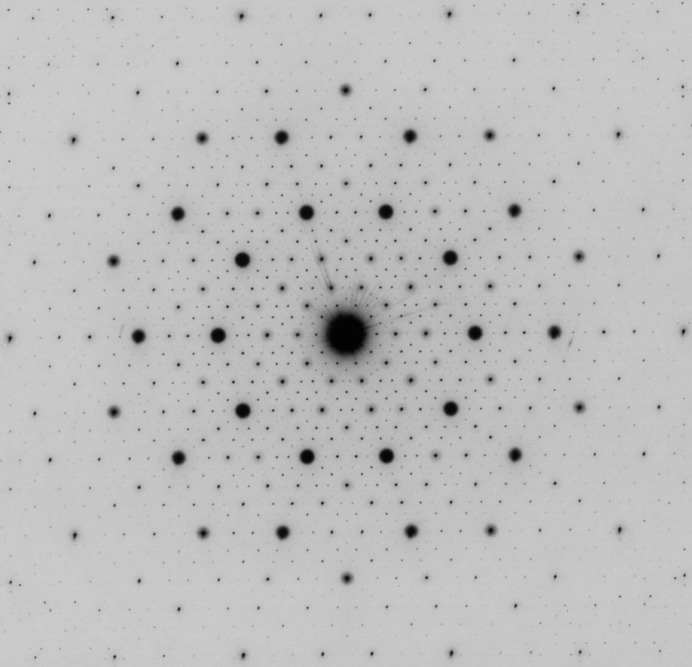
Selected-area electron diffraction pattern of an i-Sc–Zn–Mg quasicrystal with beam incidence along the fivefold axis.
